# Structure Determination and Functional Analysis of a Chromate Reductase from *Gluconacetobacter hansenii*

**DOI:** 10.1371/journal.pone.0042432

**Published:** 2012-08-06

**Authors:** Hongjun Jin, Yanfeng Zhang, Garry W. Buchko, Susan M. Varnum, Howard Robinson, Thomas C. Squier, Philip E. Long

**Affiliations:** 1 Biological Sciences Division, Pacific Northwest National Laboratory, Richland, Washington, United States of America; 2 Biology Department, Brookhaven National Laboratory, Upton, New York, United States of America; 3 Earth Sciences Division, Lawrence Berkeley National Laboratory, Berkeley, California, United States of America; Texas A&M University, United States of America

## Abstract

Environmental protection through biological mechanisms that aid in the reductive immobilization of toxic metals (e.g., chromate and uranyl) has been identified to involve specific NADH-dependent flavoproteins that promote cell viability. To understand the enzyme mechanisms responsible for metal reduction, the enzyme kinetics of a putative chromate reductase from *Gluconacetobacter hansenii* (Gh-ChrR) was measured and the crystal structure of the protein determined at 2.25 Å resolution. Gh-ChrR catalyzes the NADH-dependent reduction of chromate, ferricyanide, and uranyl anions under aerobic conditions. Kinetic measurements indicate that NADH acts as a substrate inhibitor; catalysis requires chromate binding prior to NADH association. The crystal structure of Gh-ChrR shows the protein is a homotetramer with one bound flavin mononucleotide (FMN) per subunit. A bound anion is visualized proximal to the FMN at the interface between adjacent subunits within a cationic pocket, which is positioned at an optimal distance for hydride transfer. Site-directed substitutions of residues proposed to involve in both NADH and metal anion binding (N85A or R101A) result in 90–95% reductions in enzyme efficiencies for NADH-dependent chromate reduction. In comparison site-directed substitution of a residue (S118A) participating in the coordination of FMN in the active site results in only modest (50%) reductions in catalytic efficiencies, consistent with the presence of a multitude of side chains that position the FMN in the active site. The proposed proximity relationships between metal anion binding site and enzyme cofactors is discussed in terms of rational design principles for the use of enzymes in chromate and uranyl bioremediation.

## Introduction

Contamination of groundwater, soils and sediments by long-lived soluble radionuclide wastes (e.g. uranium (U(VI))) or toxic redox-sensitive metals (e.g. chromate (Cr (VI))) from legacy of nuclear weapons development is a significant environmental problem [1]. Unfortunately, limited technologies exist to efficiently decrease the concentrations of these contaminants. An envisioned low-cost solution uses microbes to change the redox status of contaminants from soluble (e.g.: U(VI)) to insoluble species (e.g.: U(IV)). Dissimilatory metal-reducing bacteria are good bioremediation candidates given their ability to reduce iron, sulfate, chromate, or uranyl ions as a form of anaerobic respiration [2], [3]. It has been suggested that the mechanism used by these bioremediation candidates involves electron transfer reactions mediated by cytochromes located at the outer membrane or within extracellular polymeric substances (e.g., nanowires) [4], [5]. An understanding of these mechanisms has been facilitated by prior structural measurements of metal reductases (i.e., MtrC and MtrF) in *Shewanella oneidensis* MR-1, a subsurface bacterium capable of anaerobic respiration using extracellular metal oxides (e.g., Fe(III) or U(VI)) as terminal electron acceptors [6], [7]. However, while these and other dissimilatory metal-reducing bacteria have been shown to decrease U(VI) concentrations below the Environmental Protection Agency’s maximum contaminant levels (MCLs) (0.13 µM or 30 µg/L, http://water.epa.gov), relatively slow growth rates and an inability to catalyze metal reduction under aerobic conditions limit the potential of dissimilatory metal-reducing bacteria for bioremediation. In comparison, intracellular NAD(P)H-dependent FMN reductases, enzymes distributed in all bacterial species, reduce chromate or uranyl ions under both anaerobic [8], [9] and aerobic conditions [10]. These flavin-containing proteins, which include YieF (renamed ChrR) [11] and NfsA isolated from *Escherichia coli* and ChrR from *Pseudomonas putida* [10], [12], [13], have a broad substrate specificity permitting the NAD(P)H-dependent reduction of quinines, prodrugs, chromate (Cr(VI)), and uranyl (U(VI) ions [11], [12]. In the reduction of Cr(VI) to Cr (III), ChrR avoids the generation of highly toxic Cr(V), which induces oxidative stress through the production of reactive oxygen species (ROS) [14], [15].

To understand the mechanism by which intracellular NAD(P)H-dependent FMN reductases bind and efficiently reduce toxic environmental contaminants, such as CrO_4_^2−^ and UO_2_(CO_3_)_3_^4−^, we have cloned, expressed, purified, and functionally characterized a putative chromate reductase (Gh-ChrR) from the recently sequenced genome of *Gluconacetobacter hansenii* [16]. Gh-ChrR belongs to the superfamily of NAD(P)H-dependent FMN reductases that catalyze the metabolic detoxification of quinones and their derivatives to hydroquinones, using NAD(P)H as the electron donor. This family of enzymes protects cells against quinone-induced oxidative stress, cytotoxicity, and mutagenicity in both prokaryotic and eukaryotic organisms. It has been suggested that the biological role of NAD(P)H-dependent FMN reductases is to prevent futile redox cycling involving univalent reduction of diverse classes of compounds and to quench ROS [11], [12], [15]. Indeed, the overproduction of these enzymes in bacteria greatly mitigates the toxicity of pollutants such as chromate and uranyl, enhancing the ability of these bacteria to survive in environments contaminated with these compounds [11], [12]. Gh-ChrR has 57% amino acid sequence identity to *P. putida* ChrR, which has previously been shown to reduce chromate and uranyl [11], [17]. To help understand the mechanistic requirements associated with metal binding and reducing toxic heavy metals, the crystal structure of Gh-ChrR was solved at 2.25Å resolution. The structure shows that the FMN cofactor is located near subunit interfaces in a pocket containing a cationic site appropriate for binding anions (e.g. UO_2_(CO_3_)_3_^4−^ or CrO_4_^2−^) at an optimal distance for hydride transfer. Consistent with kinetic measurements, the proposed chromate binding site is near the site of putative NADH binding cleft.

## Results

### Gh-ChrR is a Flavoprotein

Recombinant Gh-ChrR was purified from *E. coli* following protein overexpression (Figure S1). The purified protein had a bright yellow color and the absorbance spectrum contained two characteristic peaks at 373 and 455 nm that indicate the presence of flavins (Figure S2). The ratio of absorbance at 267 nm to 373 nm is 2.7, suggesting that the prosthetic flavin molecule in Gh-ChrR is FMN [18], [19]. Purified Gh-ChrR contains an equimolar stoichiometry of FMN (ε_373_ = 11,300 M^−1^ cm^−1^) per monomer of Gh-ChrR (ε_280_ = 12,950 M^−1^ cm^−1^).

### NADH-dependent Metal Reduction

As expected from the sequence homology between Gh-ChrR and other members of the FMN reductase family (Pfam ID: PF03358) (Figure S3), Gh-ChrR functions as a NAD(P)H–dependent metal reductase (Figure S4, S5). Both NADH and NADPH support maximal chromate reduction by Gh-ChrR, although NADH has a higher *k_cat_*/*K_m_* than NADPH (Figure S4). This result is consistent with prior measurements where *E. coli* ChrR showed an eight-fold preference for NADH over NADPH [13]. Enzyme activity is dependent on the addition of both NADH cofactor and metal anion (e.g., chromate, ferricyanide, or uranyl) (Figure S5). Metal-dependent increases in NADH oxidation rates obey simple Michaelis-Menten kinetics (Figure S5; Table S1), permitting a simple characterization of apparent kinetic parameters linked to function. Upon NADH reduction, added metal is reduced to form Cr(III) or Ur(IV) (Figure S6). The apparent *K_m_* for uranyl is below 100 nM, which is substantially lower than previously identified for *E. coli* and *P. putida* ChrR [11], [17]. The enzyme efficiency for uranyl (*k_cat_/K_m_*>7.0×10^4^ M^−1^ s^−1^) is greater than for either chromate (1.0×10^3^ M^−1^ s^−1^) or ferricynide (1.6×10^3^ M^−1^ s^−1^). These favorable kinetic properties indicate that this enzyme may be able to efficiently decrease uranyl concentrations below the MCL.

### Substrate Inhibition Mechanism

**Figure 1:**
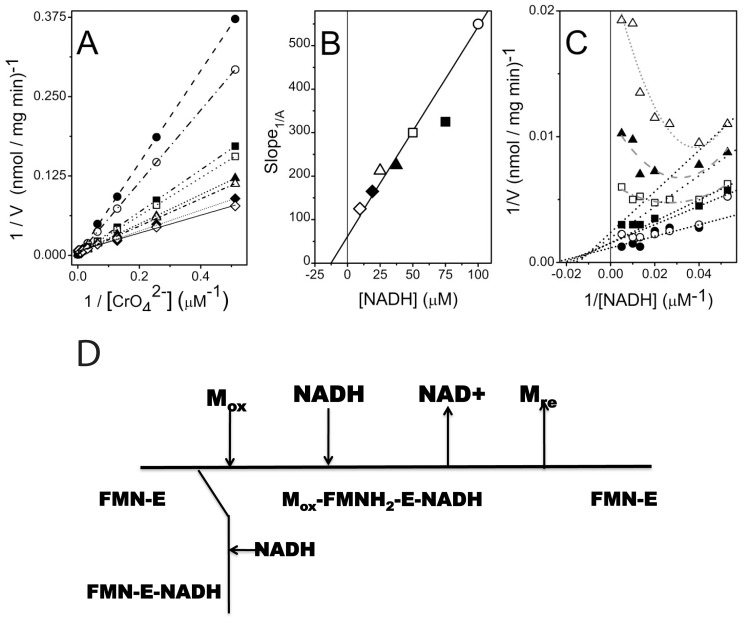
Substrate inhibition by NADH in an ordered bireactant mechanism. **A.** Double-reciprocal plots of initial velocities versus substrate concentrations assayed with fixed concentration of NADH: 9.5 µM (open diamond), 19 µM (closed diamond), 25 µM (open triangle), 37.5 µM (closed triangle), 50 µM (open square), 75 µM (closed square), 100 µM (open circle), and 200 µM (closed circle). The *V_Max_* is calculated based on the y-axis intercept on this plot. **B.** Relationship between the slopes (i.e., Slope 1/CrO_4_^2−^) in Figure 1A at each of seven fixed NADH concentrations. **C.** Double-reciprocal plots of initial velocities versus substrate concentrations with fixed concentration of CrO_4_^2−^: 31 µM (open triangle), 62 µM (closed triangle), 125 µM (open square), 250 µM (closed square), 500 µM (open circle), and 1000 µM (closed circle). At low NADH concentrations it is possible to fit the data with a straight line. However, at high NADH concentrations, individual curves bend upwards. Values for *K_mA_*, *K_mB_*, *K_ia_* and *K_i_* were calculated from axes-intercepts and slopes in panels B and C (see Table S2) [20]. **D**. Cleland notation depicting catalytic mechanism of Gh-ChrR, showing substrate inhibition by NADH binding to FMN-E to form a dead-end complex FMN-E-NADH that competes with metal complex formation, M_ox_-FMNH_2_-E-NADH.

Initial-velocity measurements with chromate as the substrate and NADH as the electron donor were carried out at a fixed enzyme concentration. Consistent with a mechanism involving substrate inhibition by NADH, there were substantial reductions in initial enzyme velocities upon increasing NADH concentrations at fixed chromate concentrations (Figure 1A). Other mechanisms, such as those involving a bi-bi ping pong reaction mechanism where increasing concentrations of NADH results in enhancements in enzyme velocity, are not consistent with the experimental data [20].

A highly characteristic relationship that is indicative of substrate inhibition is apparent when the kinetic data is plotted in the form of a double reciprocal plot comparing initial velocities relative to variable chromate concentrations at a series of fixed NADH concentrations (where NADH is the inhibitory substrate). Variable NADH concentrations only affect the slope (i.e., Slope*_1/A_*, Figure 1B), where replots of this data permit determination of additional kinetic constants (see legend to Figure 1 and supplementary data). Consistent with a mechanism of an ordered bireactant system involving substrate inhibition, a complex double reciprocal plot for fixed concentrations of CrO_4_^2−^ is observed (Figure 1C), where individual curves bend upwards at high concentrations of NADH. Collectively, these results indicate that NADH binding blocks CrO_4_^2−^ association, forming a dead-end complex (Figure 1D); Such substrate inhibition is widespread in enzymology, occurring in approximately 20% of all enzymes where mechanisms involving substrate inhibition can serve a regulatory role [21], [22].

### Structure of Gh-ChrR

**Figure 2:**
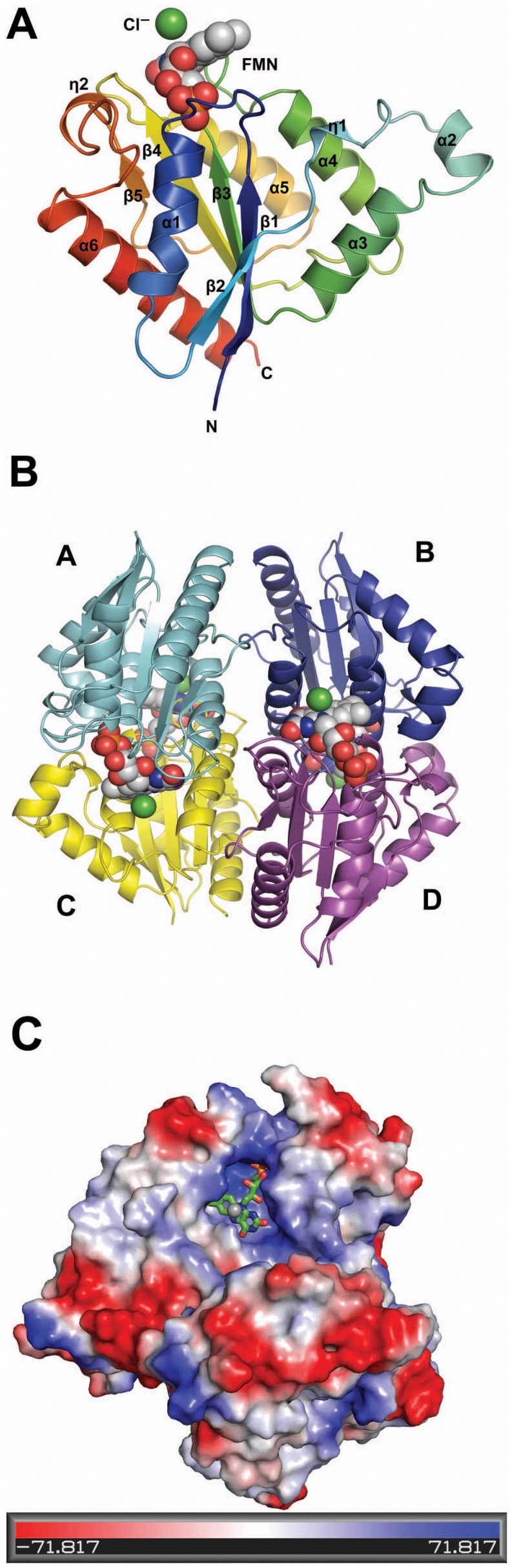
Crystal structure of Gh-ChrR. Monomeric (**A**) and tetrameric (**B**) depictions of the 2.25 Å structure of Gh-ChrR showing the backbone fold, a space-filling model of bound FMN (elements color: red  =  oxygen, blue  =  nitrogen, gray  =  carbon) and bound chloride anion (green sphere). Secondary structural elements including the 3_10_ helices (η) are numbered sequentially from the N-terminus. **C.** Electrostatic potentials at the solvent-accessible surface of Gh-ChrR. A stick model of the FMN molecule and the associated chloride ion (gray sphere) is highlighted. The electrostatic potential are drawn (Pymol) at a level of −71.817 *kT*/*e* (red) to +71.817 *kT*/*e* (blue), where *k* is the Boltzman’s constant, *T* is the absolute temperature, and *e* is the magnitude of the electron charge.

The crystal structure of Gh-ChrR was elucidated to a resolution of 2.25 Å (Table 1). The crystallographic asymmetric unit contains four monomers, each with a single bound FMN. The tetrameric structure of Gh-ChrR is consistent with the result of size exclusion chromatography (∼80 kDa), as the mass of the monomeric unit is 21.3 kDa (193 native residues plus a six residue C-terminal poly-histidine tag) (Figure S7). A tetrameric oligomerization state was also recently reported for *E. coli* ChrR [23], a protein with 61% sequence identity to Gh-ChrR. For each monomer in the asymmetric unit, electron density is missing or uninterpretable for 5–6 residues at the N-terminus and 7–9 residues at the C-terminus. Aside from the residues near the termini, there are no significant conformational differences between the four monomers as the α-carbons of residues P6-T186 superimpose on each other with a RMSD ranging from 0.35 to 0.38 Å (*UCSF-Chimeria*) [24]. Figure 2A is a cartoon representation of the backbone fold for one of the four essentially identical monomers in the asymmetric unit with the elements of secondary structure labeled. Each monomer contains two 3_10_-helices (I45-F48, V153-K156, labeled as η), six α-helices (F21-I32, Q53-E58, A62-T73, G90-R101, A125-L138, V167-T186) and five β-strands (L7-L13, I37-P40, A76-T81, P111-S118, A148-I150). The β-strands are organized into one parallel β-sheet, β2:β1:β3:β4:β5, flanked by helices α1 and α5 on one face and the remaining helices on the opposite face. The five longest helices are aligned in two groups that are approximately parallel to each other and orthogonal: (1) α1 and α3 and (2) α4, α5, and α6 so that helices are approximately parallel with each group and orthogonal between groups. Such a triple-layered, α/β/α structure resembles the fold in the flavodoxin superfamily of proteins [18], [25]. Such a triple-layered, α/β/α structure resembles the fold in the flavodoxin superfamily of proteins [19], [38] and is identical to the fold observed in the crystal structure recently reported for *E.* *coli* ChrR (PDB entry: 3SVL) [23], a structure that superimposes onto Gh-ChrR with a backbone RMSD of 0.9 Å.

**Table 1:** Data Collection and Structural Refinement Statistics of Gh-ChrR.

Data collection	
Space group	C2
Unit-cell parameters	
*a* (Å)	119.7
*b* (Å)	90.1
*c* (Å)	95.2
α = γ (°)	90.0
β (°)	119.6
Resolution (Å)	50.0–2.25 (2.33–2.25)
Redundancy	7.2 (5.6)
*I/δ(I)*	15.2 (2.5)
*R*_merge_ a	0.12 (0.50)
Completeness (%)	99.1 (91.9)
Structural refinement	
Number of reflections used in refinement	41698 (95% of total reflections)
Number of atoms Non-solvent	5604
Number of atoms Solvent	328
*R_work_* (%)^b^	19.3
*R_free_* (%)^c^	23.8
Average *B*-factor (protein)	36.04
Average B-factor (solvent)	38.05
RMSD in bond length (Å)	0.008
RMSD in bond angle (°)	1.13
Ramachandran favored (%)	98.2
Ramachandran additionally allowed (%)	1.4
Residues with bad bonds (%)	0.0

a Rmerge = Σ|Ii − <Ii>|/Σ<Ii>, where Ii is the observed intensity and <Ii> is the average intensity over symmetry equivalent measurements.

b Rwork = Σ||Fobs| − |Fcalc||/Σ|Fobs|.

c Rfree is the same as Rwork but for 5% of all reflections that were not used in crystallographic refinement (2114 reflections).

As shown in Figure 2B, the homotetramer is assembled as two sets of identical dimers (cyan/yellow and blue/purple) that are aligned side-by-side with an approximately 60 degree angle along the parallel plane α4 and α5 of each dimer. The monomer-monomer interface of each dimer is similar to that observed in other flavodoxin-like dimers, such as *P. aeruginosa* T1501 [26] or *Saccharomyces cerevisiae* Ycp4 [27]; the homotetramer structure is similar to the assembly observed in some other FMN reducatases, including ChrR from *E. coli* [23], EmoB from *Mesorhizobium* sp. BNC1 [28], ArsH from *Shigella flexneri* [18] and *Sinorhizobium meliloti* [25]. In Gh-ChrR this monomer-monomer interface is composed primarily of α5, α4, and the loop between β3 and α4, and to a lesser degree of α2, η1, and the loop between β1 and α1. In turn, the dimer-dimer interface is composed primarily of α5 and the loop between α5 and β5. The accessible surface area of the Gh-ChrR tetramer is 25360 Å^2^ and the buried surface area is 13620 Å^2^ (53.9%) or 3405 Å^2^ per monomer. This is substantially greater than the mean buried surface area for the dimer-dimer interface, 2640 Å^2^ (18%) or 1320 Å^2^ per monomer.

### FMN Binding Site

**Figure 3:**
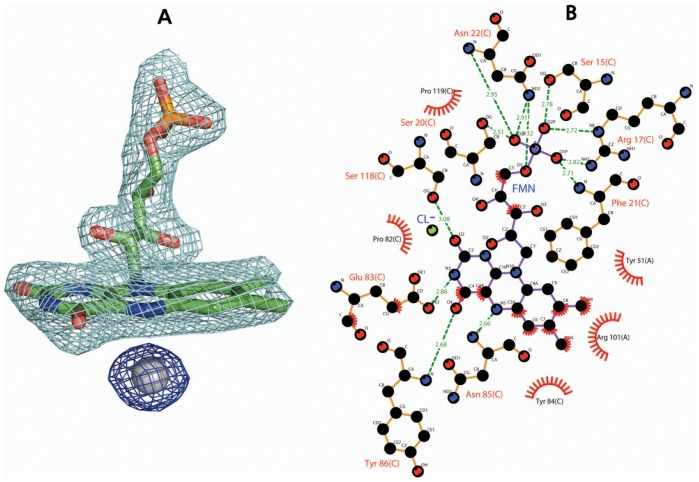
Structure proximal to bound FMN. **A.** Electron density surrounding FMN and chloride ion (gray sphere) contoured at 1.0 σ. **B.** Schematic representation of hydrophobic contacts (arc with radiating spokes) and potential hydrogen bonds (dashed lines) between FMN and two monomeric units (chain A and C) of the Gh-ChrR tetramer. Atoms are color-coded: black = carbon, red = oxygen, blue = nitrogen. This image was produced using the program *LIGPLOT* [62].

As shown in Figure 2A and 2B, Gh-ChrR crystallized with one molecule of FMN associated with each protein monomer. FMN binds in a pocket on the surface of Gh-ChrR near the dimer interface. The FMN binding pocket is more clearly illustrated in Figure 2C, which highlights the electrostatic potential at the solvent-accessible surface of Gh-ChrR. The negatively charged ribityl phosphate group of FMN is deeply buried in a positively charged region (blue) composed of residues in the loop between β1 and α1 (S15–N22) and a positive electrostatic dipole from the N-terminus of the capped α-helix (α1) similar to that previously reported [29]. On the other hand, the aromatic isoalloxazine ring sits in a more hydrophobic region (white) of the binding pocket. Details of the protein-FMN contacts responsible for stabilizing the complex are shown schematically in two-dimensions in Figure 3: 12 hydrogen bonds and five hydrophobic contacts. Except for two hydrophobic contacts (Y51’ and R101’), the FMN-protein interactions at the dimer interface are with one monomeric unit. As a consequence, the active site of Gh-ChrR is open and solvent accessible, a feature observed at the active site of oxidoreductases that facilitates promiscuous exchange of substrates [30]–[32].

Flavodoxins are commonly identified in genomes by primary amino acid sequence analysis and a fingerprint FMN-binding motif, T/SXTGXT, responsible for binding to the ribityl phosphate group [33], [34]. In Gh-ChrR the equivalent sequence for this motif is G^14^SLRKASFN^22^. The sequence for this region in Gh-ChrR is similar to some other NAD(P)H-dependent FMN reductases including *E. coli* ChrR [23] (PDB entry: 3SVL) and two flavoproteins (PDB entries: 1NNI and 2VZY) shown to form tetramers [28], [35] (Figure S3). Within this FMN-binding sequence the side chains of the Gh-ChrR residues that make specific contacts with the ribityl phosphate group, S15, R17, S20, and N22, are conserved among the aligned sequences. It is worth noting that the amino acid sequence of the region responsible for binding to the isoalloxazine ring in Gh-ChrR, P^82^EYNY^86^, is conserved (Figure S3).

### Putative NADH Binding Site

**Figure 4:**
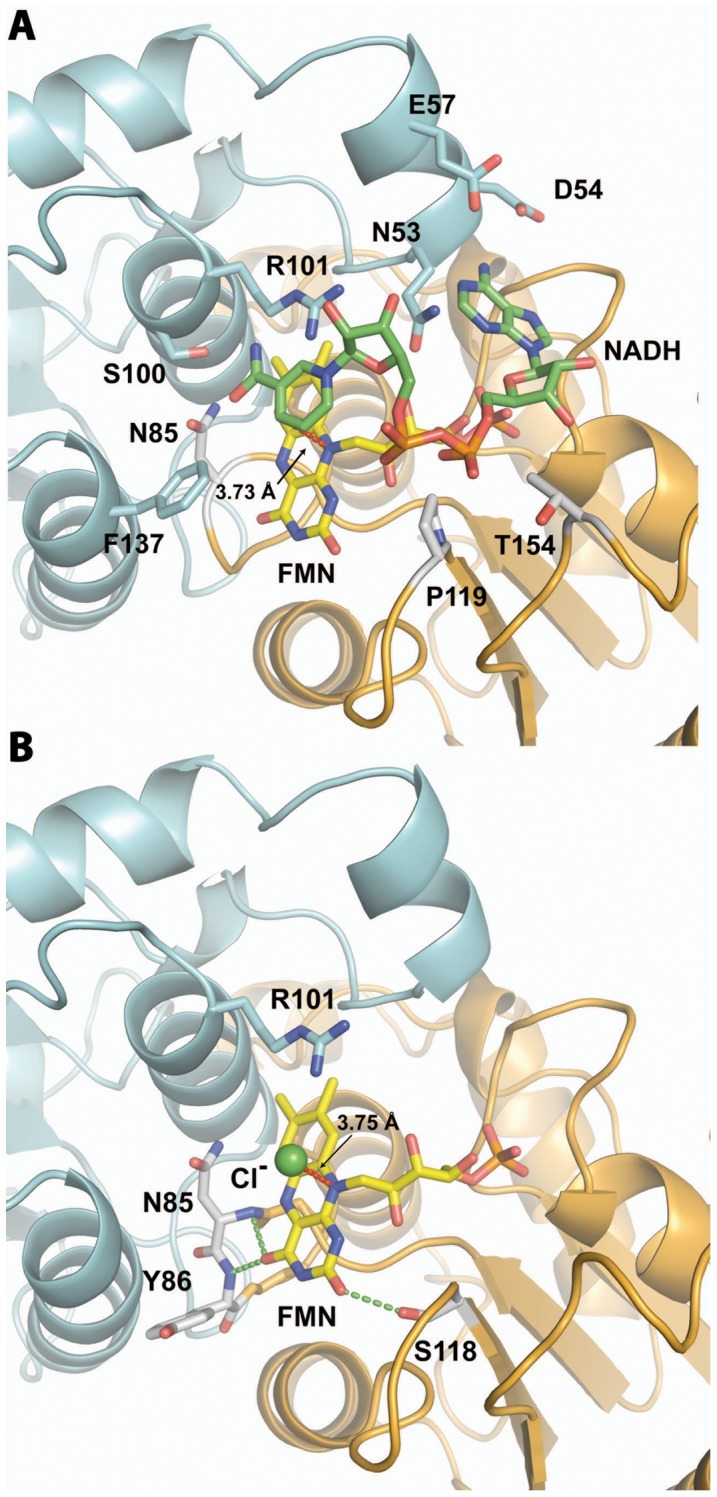
Putative Gh-ChrR NADH and substrate binding sites. **A.** NADH was modeled into the Gh-ChrR structure by superimposing it with the NADH-containing structure of EmoB (PDB entry: 2VZJ, Figure S7). The nicotinamide ring of NADH (primarily green stick model) is stacked on top of the isoalloxazine ring of FMN (primarily yellow stick model), and the adenosine part of NADH points to ribtyl group of FMN. The black arrow indicates the distance from C4N of NADH to the *si*-face of the FMN isoalloxazine ring. Residues N53, D54, E57, S100, R101 and F137 from chain A (cyan) and residues N85, P119, and T154 from chain C (gold) interact with NADH. **B.** The putative active site of Gh-ChrR shown with bound FMN (primarily yellow stick model) and a chloride ion (green sphere). The black arrow indicates the distance from the Cl^−^ to the *si*-face of the FMN isoalloxazine ring. Key residue R101 holding chloride ion in place is shown in a stick model. Critical residues for hydride transfer, N85 and Y86 from chain A (cyan) and S118 from chain C (gold) are shown in a stick model. The green dash lines indicate the distance (∼3 Å) between N of amide group of N85/Y86 and O4, and the distance (∼3 Å) between OG of hydroxyl group of S118 and O2.

While bound FMN is observed in the crystal structure of Gh-ChrR (Figures 2 and 3), NADH, an essential electron transfer component of the reductive reactions catalyzed by NAD(P)H-dependent FMN reductases, is absent. Efforts to co-crystallize Gh-ChrR with NADH were unsuccessful. However, it is possible to predict the location of the NADH binding site on the FMN-Gh-ChrR structure by superposing it on the structure of a homologous NAD(P)H-dependent FMN reductase, EmoB from *Mesorhizobium* BNC1 complexed with FMN and NADH [28]. Both Gh-ChrR and EmoB form homotetramers that have similar structures for the individual monomeric subunits (RMSD =  2.6 Å with 161 aligned Cα atoms, Figure S8). In EmoB, the nicotinamide ring of NADH sits above the bound FMN and stacks against the isoalloxazine ring of FMN. Only two residues in EmoB were observed to interact with NADH, K81 and G112 [28]. In the superimposition with Gh-ChrR (Figure 4A and Figure S8), only one of the two equivalent residues, N85, is in a position to contact NADH, as G109 is too distant. The importance of N85 was confirmed by an N85A site-directed substitution (Table 2), resulting in an apparent *K_m_* value 3-fold larger than that for wild type Gh-ChrR that is consistent with a reduction in binding affinity. The aromatic ring of F137 is 2.82 Å from C4N of the NADH nicotinamide ring suggesting a possible hydrophobic interaction. Other Gh-ChrR residues that could potentially interact with NADH are N53, D54, and E57 at the adenosine part of NADH and P119 and T154 at the di-phosphate part of NADH. Collectively, the superposition of structures suggests that residues N53, D54, E57, S100, R101 and F137 from one monomer and residues N85, P119, and T154 from the other monomer of the dimer, may interact with NADH (Figure 4A), and further suggests that the active site of Gh-ChrR has ample room for NADH to enter.

**Table 2:** Catalytic Influence of Site-Directed Substitution of Putative Metal and Cofactor Ligands on NADH-Dependent Chromate Reduction Efficiency.

Gh-ChrR variants	V_max_ [nmol/(min mg enzyme)]	Apparent *K_m_* (µM)^a^	Apparent *k_cat_* (s^−1^)^a^	Apparent *k_cat_/K* (M^−1^ s^−1^)^b^
wt	750±90	240±70	25±3×10^−2^	1.0±0.3×10^3^
S118A	900±100	600±20	30±4×10^−2^	0.5±0.2×10^3^
N85A	160±20	600±100	5.3±0.5×10^−2^	0.09±0.02×10^3^
R101A	240±50	1900±600	8±2×10^−2^	0.04±0.01×10^3^

a Values are based on triplicate measurements, where steady-state kinetic data for Gh-ChrR (5 µM) were measured at a constant NADH concentration (100 µM) and fit to the Michaelis-Menten equation.

b Calculations based on a molecular mass of tetrameric Gh-ChrR, 80kDa, and four independent active sites.

### Putative Metal Anion Binding Site

Based on the substrate inhibition mechanism (Figure 1), metal is reduced only if it binds to Gh-ChrR before NADH. If NADH binds to Gh-ChrR before the metal a dead-end product forms that blocks metal binding. Unless NADH binding induces significant structural changes to Gh-ChrR upon binding, this sequence suggests that the substrate (metal) binding site is near the tightly bound FMN molecule. Attempt to co-crystallize Gh-ChrR bound to either chromate or uranyl was unsuccessful along with attempts to form complexes by soaking Gh-ChrR crystals with chromate or uranyl. However, spherical electron density was observed on the *si*-face of the FMN isoalloxazine ring (Figure 3A) in a similar position observed for FMN in BluB from *S. meliloti* [32]. In BluB this electron density was modeled as molecular oxygen, but for Gh-ChrR it is best fit with a Cl^-^ ion because of its spherical rather than ellipsoidal shape. The distance from the plane of the isoalloxazine ring is similar in Gh-ChrR and BluB, 3.7 and 3.5 Å, respectively. This ligand-isoalloxazine ring distance is likewise similar in some other NAD(P)H-dependent FMN reductases with bound ligands such as CrS from *Thermus scotoducutus* SA-01 (PDB entry: 3HF3) [36] and WrbA from *E. coli* (PDB entry: 3BK6) [37], where the ligand-FMN distance is 3.38 and 3.40 Å, respectively. It is not immediately apparent what forces are holding the heteroatom in place because the nearest positively charged counter ion is the 4.57 Å distant amide group of the side chain of R101 (Figure 4B). By contrast, in the crystal structure of *T. scotoducutus* CrS SA-01 (PDB entry: 3HF3) [36] the sulfide ion on the *si*-face of the FMN isoalloxazine ring is held in place by the side chains of two histidine residues less than 3 Å away. Regardless of the forces holding a negatively charged Cl^−^ ion in place in Gh-ChrR, the side chain of R101 is a good candidate to assist the binding of an anion (Figure 4B). The importance of R101 in metal binding and NADH interaction was confirmed by kinetic studies on a Gh-ChrR construct containing a R101A substitution. The enzyme efficiency (apparent *k_cat_*/*K_m_* value) of R101A for chromate was 25 fold less than that for wild type Gh-ChrR, and the apparent *K_m_* value was 8 fold greater than that for wild type Gh-ChrR (Table 2). Negatively charged species analogous to Cl^−^, including chromate, ferricyanide and uranyl ions, may be recruited to the catalytic center about R101 at a favorable distance (∼3.5 Å) for hydride transfer from FMNH_2_ to the metal [38].

## Discussion

This study demonstrates that recombinant Gh-ChrR has the ability to reduce metal oxides (chromate, ferricyanide, uranyl) (Figure S5). Of particular interest, Gh-ChrR binds and reduces uranyl with a higher affinity (apparent *K_m_*<100 nM; Table S1) than any other enzyme in this class [11], [13], [17]. The mechanistic basis for high-affinity binding and reduction of bound metal oxides can be understood from the 2.25 Å crystal structure of Gh-ChrR (Figure 2, 3 and 4). Proximal to the FMN binding pocket near the subunit interface, a cationic cleft is observed that has optimal geometrical properties to bind NADH and either chromate or the physiologically relevant UO_2_(CO_3_)_3_^4−^ anion present at contamination sites (Rifle, CO) [39], [40], permitting efficient enzyme cycling (Figure 1).

As observed in the structure of other flavodoxins [18], [25], [26], [28], the cofactor FMN in Gh-ChrR is non-covalently bound near the dimer interface and held in place primarily via contacts with α5, α4, and the loop between β3 and α4. Enzyme kinetic measurements suggest that chromate and NADH bind sequentially to Gh-ChrR at different sites that are consistent with the presence of a positively charged groove in the catalytic pocket near the FMN (Figures 3 and 4). In the crystal structure a negatively charged chloride ion is observed bound in this pocket above the *si*-face of the isoalloxazine ring of FMN where the negatively charged chromate (CrO_4_^2−^), ferricyanide (Fe(CN)_6_^3−^), or uranyl (UO_2_(CO_3_)_3_^4−^) species may bind in a similar manner. If NADH binds to this metal binding site first, a dead-end product results and metal reduction is inhibited (Figure 1). If chromate, ferricyanide, or uranyl binds to this metal binding site first, NADH can move into its proper binding site in the positively charged groove at an optimal distance (∼3.7 Å) for hydride transfer. Note that the analysis of the crystal structure of Gh-ChrR in the absence of substrates suggests that the metal and NADH binding sites overlap: NADH binds on top of the ribityl group and the isoalloxazine ring of FMN and the metal binds on top of the isoalloxazine ring of FMN (Figure 4). Binding of the metal anion may induce some structural rearrangement in the active site of Gh-ChrR to remove this overlap and allow both species to bind simultaneously. After the chromate (VI) or uranyl (VI) ions are reduced to less soluble chromium (III) and uranium (IV) species, respectively, they are released from the catalytic center of the enzyme into the solvent. The open, solvent accessible nature of the catalytic pocket in Gh-ChrR may facilitate binding and reduction of a broad spectrum of substrates. There is no exchangeable proton, at least in the crystal structure of Gh-ChrR with oxidized FMN, near enough to stabilize the negative charge at N1 in the semiquinone form of FMN. The closest atoms to N1 that may serve as a general acid/base catalyst for protonation/deprotonation are the hydroxyl group of S118. The importance of S118 in catalysis was corroborated by kinetic studies on individual Gh-ChrR constructs containing a S118A substitution. The chromate reduction assay revealed that the catalytic efficiency (apparent *k_cat_*/*K_m_* value) of S118A was 50% reduced compared to wild type Gh-ChrR (Table 2).

The mechanism of two-electron reduction of U(VI) to U(IV) is straight-forward, it becomes more complicated for the odd electron reduction of Fe(III) to Fe(II) and Cr(VI) to Cr(III) and likely involves transferring of electron(s) to molecular oxygen and the generation of ROS [11]. This was confirmed by experiments designed to measure ROS generation that showed chromate and ferricyanide reduction produced 5–6 times more ROS than uranyl reduction (Figure S9).

While extracellular electron transport for metal reduction or detoxification has been shown to be effective under field conditions [41], [42], metal reduction by cytosolic enzymes may provide an alternative reduction pathway. Based on our kinetic measurements, Gh-ChrR reduces highly soluble chromate, ferricyanide, and uranyl oxides to a less soluble reduced state using NADH as the electron donor. Optimal uranyl reduction is observed using a carbonate buffer that approximates subsurface conditions, which are dominated by negatively charged aqueous complexes of U(VI) such as UO_2_(CO_3_)_3_^4−^ [39], [43]. This suggests that Gh-ChrR may be a useful enzyme for uranium bioremediation in aquifers.

Many toxic metals and radionuclides can be precipitated and immobilized naturally by bacterial bioreduction. This phenomenon has been widely investigated as a promising, inexpensive approach for bioremediation of radionuclide and heavy metal contaminants [1], [44]. Dissimilatory sulfate-reducing bacteria and dissimilatory ion-reducing bacteria have received intense attention as their extracellularly-located respiration chain can catalyze the desired reactions. However, respiration involving extracellular reactions is subject to inhibition by nitrate and oxygen, which commonly occur at contaminated sites, requiring terminal electron-accepting processes (TEAPs) to remove theses constituents before metal-reducing TEAPs can be initiated. In addition, reduced species formed in the extracellular environment, may be reoxidized [45]–[47]. Alternative approaches involving metal precipitation inside cells are promising and could be used to supplement extracellular processes or possibly as the primary enzymatic reductive process [48]. From this perspective, the ability of intracellular enzymes such as Gh-ChrR to catalyze the reduction of uranyl under aerobic conditions may lead to novel strategies for bioremediation of U(VI) in groundwater. Although *G. hansenii* is not a bacterium usually found at the environmental subsurface, recombinant Gh-ChrR has a capability of reducing chromate and uranium under aerobic condition in the micromole range, and therefore, may be a useful protein for bioremediation bioengineering (e.g. immobilized on bacteriophage or nanoparticle surfaces).

## Materials and Methods

### Chemicals and Reagents

Ni-NTA affinity resin was purchased from Qiagen Inc. (Valencia, CA), isopropyl β-D-1-thiogalactopyranoside (IPTG) and LB medium from Fisher Scientific (Wilmington, MA), and the crystallization screening kits from Hampton Research (Aliso Viejo, CA) and Emerald BioSystems (Bainbridge Island, WA). Uranyl (U(VI)) acetate dihydrate was purchased from Fluka (now Sigma-Aldrich Fine Chemicals, St. Louis, MO). All other chemicals were purchased from Sigma-Aldrich Fine Chemicals (St. Louis, MO).

### Protein Expression and Purification

The DNA sequence of the Gh-ChrR gene from *Gluconacetobacter hansenii* ATCC 23769 (ZP_06834583) plus three site directed mutations (S118A, N85A and R101A) was codon optimized for expression in *E. coli*, synthesized, and inserted into the expression vector pJexpress411 (DNA 2.0 Inc., Menlo Park, CA, USA) such that a 6-histidine tag was present at the C-terminus of the gene product. The recombinant plasmid was then transformed into the *E. coli* expression host BL21(DE3). A single colony from a selection plate was inoculated into 20 mL of LB medium containing 40 µg/mL kanamycin. Following overnight incubation at 37°C this culture was transferred into 1 L of LB medium containing 40 µg/mL kanamycin and further incubated at 37°C until an OD_600_ of 0.8–1.0 was reached. Protein expression was then induced by the addition of IPTG to the medium (0.02 mM final concentration). The temperature was immediately lowered to 14°C and 16 h later the cells were harvested by centrifugation and frozen at −80°C.

To purify Gh-ChrR, thawed cells were first resuspended in lysis buffer (50 mM K_2_HPO_4_-NaH_2_PO_4_, 300 mM NaCl, pH 8.0), sonicated (Branson Ultrasonic, Danbury, CT) for 1 min three times on ice, and centrifuged at 10000×g at 4°C for 20 min to remove cellular debris. The supernatant was collected and incubated with Ni-NTA resin at 4°C for 1 h. The mixture was then loaded into an empty column and washed sequentially with lysis buffer containing increased concentrations of imidazole. Gh-ChrR was eluted using lysis buffer containing 200 mM imidazole. This eluent was concentrated to ∼1 mL (Millipore Amicon Centriprep) prior to loading onto a 1 mL Hitrap Q ion exchange column (GE Healthcare, Piscataway, NJ) connected to an ÄKTA explorer FPLC system (GE Healthcare, Piscataway, NJ) for further purification. Using a 0 to 1 M NaCl linear gradient, a major band containing Gh-ChrR eluted at a NaCl concentration between 0.15–0.2 M. Purified Gh-ChrR, which was yellow in color, was concentrated for structural and functional analyses. The SDS-PAGE analysis of the final product showed the sample to be >95% pure (Figure S1).

### Reductase Activity Assays

Measurements of absorbance changes for NADH (ε_340_ = 6220 M^−1^ cm^−1^) accurately measure the metal-dependent reductase activity of the chromate redutase enzyme ChrR, as previously validated by Puzon and coworkers [8]. Interference from the absorbance of chromate is minimal despite the absorbance shoulder of CrO_4_^2−^ at 340 nm (900 M^−1^ cm^−1^) due to the apparent isosbestic point of Cr(III) at 340 nm, resulting in minimal interference [49]. As a result, measurements of NADH oxidation are routinely used to measure the function of chromate reductases, in both the absence of chromate as originally referenced [18], [35], and in the presence of chromate [8], [15]. All measurements are consistent with standard assays using 1,5-diphenylcarbazide [50], which were used in a limited number of measurements to validate observed experiments. Likewise, neither U(IV) or U(VI) have significant absorption at 340 nm [51], [52], allowing measurements of NADH reduction rates to assess enzyme activity.

The ability of Gh-ChrR to reduce Cr(VI), Fe(III), and U(VI) was assayed using 96-well microplates by measuring NADH consumption using the absorbance at 340 nm (A_340_, ε = 6220 M^−1^ cm^−1^) [8], [15]. Initial velocity measurements for the reduction of Cr(VI) and Fe(III) were carried out at 37°C using various concentrations of potassium chromate and potassium ferricyanide in a 100 µL assay buffer (50 mM Tris-HCl, 100 mM NaCl, pH 7.4) containing 100 µM NADH and 5 µM Gh-ChrR. For reduction of U(VI), the assay was similar with that for Cr(VI) and Fe(III), except the assay buffer contained 100 mM NaHCO_3_-Na_2_CO_3_, 50 mM NaCl, pH 8.3. This assay buffer helps uranyl form stable negatively charged coordinated species, as previously reported [39], [53]. The kinetic experiments were all conducted by adding metal anions before the NADH. All kinetic data were measured on a SpectraMax 384Plus microplate reader (Molecular Devices, Sunnyvale, CA). Values for apparent *K_m_* and *V_max_* were calculated by fitting the data to Michaelis-Menten plots using KaleidaGraph version 4.0 (Synergy Software, Reading, PA) (Table S1). All measurements were conducted in triplicate under aerobic conditions.

### Substrate Inhibition Studies

Chromate is reduced by Gh-ChrR in an NADH and chromate dependent manner. Increased concentrations of NADH lead to a reduction in the enzyme velocity of Gh-ChrR, suggesting that NADH may bind to the enzyme form a dead-end complex (Figure 1). Consistent with this observation, prior data has demonstrated an inhibition of metal reductase activity at elevated NADPH concentrations for ChrR from *Thermus scotoductus* SA-01 [54]. Such a report is consistent with data for a similar NADPH-dependent quinone oxidoreductase enzymes from *E. coli* that also show substrate inhibition mechanisms [55]. To understand whether NADH acts as a substrate inhibitor [21], a mechanism involving substrate inhibition using an ordered bireactant model was investigated [20], where:


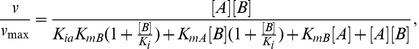
(1)

Terms in the equation are defined as:

*v* is the initial velocity,

*V_max_* is the maximum velocity,

*[A]* is the concentration of CrO_4_^2−^,

*[B]* is the concentration of NADH,

*K_ia_* is the dissociation constant of CrO_4_^2−^,

*K_mB_* is the Michaelis-Menten constant of NADH,

*K_i_* is the dissociation constant of the dead-end product of FMN-Gh-ChrR-NADH,

*K_mA_* is the Michaelis-Menten constant of CrO_4_^2−^

When the noninhibitory substrate (i.e., CrO_4_^2−^ or A) is varied, the equation simplifies to:


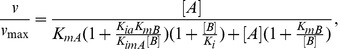
(2)

Double reciprocal plots (i.e., *1/v* versus *1[A]*) result in a family of lines with varying slopes (i.e., Slope*_1/A_*) at fixed concentrations of the inhibitory substrate (i.e., [NADH] or [B]). There is a common intersection on the y-axis upon extrapolation to infinite B that corresponds to *1/V_max_* (Figure 1A). Relationships between the slopes (i.e., Slope*_1/A_*) in Figure 1A at fixed NADH concentrations provide additional kinetic information (Figure 1B), since the replot of slope1/A is given by:


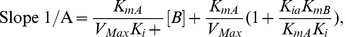
(3)

where the slope of the replot equals *K_mA_/V_max_ K_i_*, the y-intercept corresponds to:


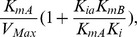


and the x-axis intercept is:


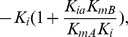


Additional information is available from a consideration of the double reciprocal plot of velocity versus the concentration of the inhibitory substrate (i.e., [NADH] or [B]) at fixed concentrations of the noninhibitory substrate (i.e., [CrO_4_^2−^] or [A]). This plot (Figure 1C) yields a family of curves that demonstrate significant curvature at high concentrations of NADH (i.e., low values of 1/[NADH]. Under these substrate conditions, the velocity equation is:


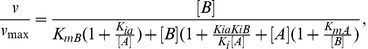
(4)

A linear relationship is only observed at low concentrations of NADH (i.e., high values of 1/[NADH]). Extrapolation of the linear curves results in a family of curves that intersect at a common point (x, y) to the left of the y-axis, where x equals:


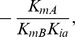


.and y equals:


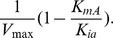


Simultaneous solutions to the above equations permit calculation of the kinetic parameters *K_mA_, K_mB_, K_ia_* and *K_i_* (Table S2).

### Cr**(**III**)** and U**(**IV**)** Determination

The Cr(III) product was measured by the absorbance at 580 nm, as previously reported [8], [13]. The determination of U(IV) levels was based on the chemical reduction of Fe^3+^ to Fe^2+^ by U(IV) and the subsequent reaction of Fe^2+^ with 1,10-phenanthroline to produce a red/orange color with an absorbance at 510 nm [11]. The latter assay involved the addition of 100 µL of a colorimetric solution consisting of FeCl_3_ (1 mM, pH 2.0), 1,10-phenanthroline (10 mM), and sodium acetate (1 M, pH 4.0) in a 5∶1:1 volumetric ratio to each reaction well. The relative U(IV) concentration was determined using a standard curve prepared with different concentrations of FeSO_4_.

### Size Exclusion Chromatography

The oligomeric state of Gh-ChrR in solution was determined by the retention time obtained for freshly prepared Gh-ChrR (from the HiTrap Q column and buffer exchanged into 50 mM Tris-HCl, 100 mM NaCl, pH 7.4) at the same elution condition. This involved the use of a Superdex 75 10/30 column (GE Healthcare, Piscataway, NJ) pre-equilibrated with the elution buffer (50 mM Tris-HCl, 100 mM NaCl, pH 7.4) and calibrated using three molecular mass standards (ribonuclease A (13.7 kDa), ovalbumin (44 kDa), and conalbumin (75 kDa)) (GE Healthcare, Piscataway, NJ) with a flow rate of 0.3 mL/min. The oligomeric state of Gh-ChrR in solution was then determined by the retention time obtained for freshly prepared Gh-ChrR.

### Crystallization and Structure Determination

The Gh-ChrR solution used for the kinetic experiments was buffer exchanged into 20 mM Tris-HCl, 150 mM NaCl, pH 7.4 and concentrated to ∼9 mg/mL for the crystallization trials. The hanging-drop vapor diffusion method was used for crystallization at room temperature by mixing 2 µL of protein solution with 2 µL of precipitant and equilibrating against 500 µL of precipitant. Crystals suitable for X-ray data collection were obtained with precipitant containing 0.5% of PEG4000, 10% isopropanol, and 0.1 M HEPES (pH 7.5). These crystals were incubated stepwise into cryoprotectant solutions with increasing concentrations of glycerol (up to 30%) prior to flash freezing in liquid nitrogen. X-ray data collection was performed with an ADSC Q315 CCD detector at beamline X29A at the National Synchrotron Light Source (NSLS) at Brookhaven National Laboratory. Diffracted data were processed using *DENZO* and integrated intensities were scaled using *SCALEPACK* from the *HKL-2000* program package [56]. The structure of Gh-ChrR was phased by molecular replacement using *Phaser* from the *CCP4* suite [57] and the crystal structure of a FMN reductase from Pseudomonas aeruginosa PA01 (PDB entry: 1RTT) [26] as the search model. The side chains of non-conserved residues between the model and Gh-ChrR were truncated using the *CHAINSAW* program. Molecular replacement yielded an initial structure with *R_work_* of 0.431 and *R_free_* of 0.442. One round of rigid body refinement and restrained refinement using the REFMAC program from the *CCP4* suite reduced the *R_work_* and *R_free_* to 0.328 and 0.375, respectively, indicating the molecular replacement was successful. The missing side chains were manually rebuilt using the Crystallographic Object-Oriented Toolkit (*Coot*) [58] followed by numerous iterative rounds of restrained refinements using *REFMAC*. Improvement of structure quality was monitored by the decrease of *R_work_* and *R_free_* after each round of *REFMAC* refinement. The final Gh-ChrR model yielded a R_work_ of 0.193 and a R_free_ of 0.238 and the stereochemistry of the final structure was assessed by *MOLPROBITY* [59]. Detailed data collection and structural refinement statistics for Gh-ChrR is listed in Table 1.

### Structure Analysis and Modeling

Except where specifically noted, all figures of protein structures were generated using PyMOL [60]. The Gh-ChrR structure was superposed with the EDTA monooxygenase B(EmoB)-NADH complex (PDB entry: 2VZJ) using the UCSF-Chimera *MatchMaker* program by aligning the Cα atoms of Gh-ChrR (PDB entry: 3S2Y) with those of EmoB [24]. The lowest RMSD (root mean square deviation) solution was selected for further analyses.

### ROS Measurement

ROS generation during the reduction of chromate, ferricyanide and uranyl by the Gh-ChrR was measured using the oxidation-sensitive fluorescent probe 5-(and-6)-carboxy-2′, 7′-dichlorodihydrofluorescein diacetate (Invitrogen; Grand Island, NY) following the standard protocol [61]. Briefly, the probe was dissolved in DMSO to a stock concentration of 200 µg/mL and 10 µl this solution was added to 100 µL of reaction buffer containing 5 µM enzyme, 500 µM substrate, and 100 µM NADH. After incubation at 37°C for 30 min, the steady state fluorescence was measured using a 96-well SpectraMax GenMiniXS reader (Molecular Devices, Sunnyvale, CA), with excitation wavelength at 488 nm and emission wavelength at 535 nm. The mean fluorescence intensity of eight reaction trials, with or without Gh-ChrR (5 µM), was determined. Background fluorescence was measured in the absence of either metals or NADH, and generally was less than 500 Arbitrary Fluorescence Units (AFU).

## Supporting Information

Figure S1 **SDS-PAGE analysis of the HiTrap Q fractions containing recombinant Gh-ChrR.** Lane1: Molecular weight markers (labeled in kDa on the left). Lanes 2–9: Two µL aliquots from sequential fractions off a HiTrap Q ion exchange column. The major band identified with an arrow is Gh-ChrR (monomeric molecular mass  = 21.3 kDa). Fraction 5, free of visible impurities on the gel, was used to crystallize Gh-ChrR and for the enzyme kinetic studies.
(TIF)

Figure S2 **Influence of NADH on the UV/vis absorbance spectrum of Gh-ChrR.** Spectra of a freshly purified solution of Gh-ChrR (15 µM protein, 50 mM Tris-HCl, 100 mM NaCl, pH 7.4) recorded before (solid line) and after (dashed line) the addition of excess NADH (100 µM). The spectral changes could be observed visually with the original yellow colored sample turning clear upon the addition of NADH indicating a transition from an oxidized to a reduced state.
(TIF)

Figure S3 **Sequence alignment of Gh-ChrR with other NAD(P)H-dependent FMN reductases** (**NDFR**)**.** The amino acid sequences of the following FMN reductases were aligned using ClustalW2 (http://www.ebi.ac.uk/Tools/msa/clustalw2/): **Gh_NDFR**: *G. hansenii* Gh-ChrR [16]; **Ec_NDFR**: *E. coli*. IAI39 YieF [63]; **Pa_NDFR**: *P. aeruginosa* PAO1 NDFR reductase [26] (PDB entry: 1RTT); **Bc_NDFR**: *B. subtilis str.* 168 NDFR [35] (PDB entry: 1NNI); **Edb_NDFR**: EDTA-degrading bacterium BNC1 EmoB [28] (PDB entry: 2VZJ); **Cpa_NDFR**: *Candidatus Protochlamydia amoebophila* UWE25 NDFR [64]; and **Rp_NDFR**: *Rhodopseudomonas palustris* NDFR [65]. The secondary structure elements of Gh-ChrR are indicated on the top sequence (3_10_ helices are indicated as η). Identical and conserved residues are highlighted red and yellow, respectively. The elements of secondary structure observed in the crystal structure of Gh-ChrR are shown on top of the alignment.
(TIF)

Figure S4 **Influence of NADH and NADPH on the rates of chromate reduction by Gh-ChrR.** Rate of chromate reduction by Gh-ChrR (5 µM) in the presence 200 uM NADH (open circles) and 200 µM NADPH (open triangles) in buffer containing 50 mM Tris-HCl, 100 mM NaCl, pH 7.4. Experiments were performed in triplicate with error bars for each measurement shown.
(TIF)

Figure S5 **Reduction of chromate, ferricyanide, and uranyl by Gh-ChrR.** NADH-dependent reduction rates and associated nonlinear least squares fits (solid lines) for Gh-ChrR (5 µM) in the presence of the indicated concentrations of the metal oxides chromate (**A**), ferricyanide (**B**), and uranyl (**C**). Measurements were made by following NADH consumption and represent the average of triplicate experiments. The kinetic parameters obtained from a nonlinear least-squares fit of this data to Michaelis-Menten equations are listed in Table S1.
(TIF)

Figure S6 **Increase in the levels of Cr(III) and U(IV) following the reduction of Cr(VI) and U(VI), respectively, by Gh-ChrR. A.** The chromate reduction product Cr(III) was monitored by the increase in absorbance at 580 nm observed by incubating 125 µM Cr(VI) and 100 µM NADH with (open circles) and without (open triangles) Gh-ChrR. **B.** The uranyl reduction product uraninite (U(IV)) was monitored by the increase in absorbance at 510 nm observed by incubating 125 µM U(VI) and 100 µM NADH with (white column) and without (grey column) Gh-ChrR. Shown are the average of three independent measurements recorded 20 minutes after the addition of the protein.
(TIF)

Figure S7 **Determination of the native molecular weight of Gh-ChrR by size exclusion chromatography.** **A.** Stacked elution profiles (monitored absorbance at 280 nm) of Gh-ChrR (bottom) and molecular weight standards on a Superdex75 size exclusion column (50 mM Tris-HCl, 100 mM NaCl, pH 7.4): 1. ribonuclease A (13.7 kDa, top panel); 2. ovalbumin (44 kDa, middle panel); and 3. conalbumin (75 kDa, middle panel). The dashed line in the bottom chromatogram is the absorbance at 340 nm corroborating that FMN was bound to the protein. **B.** The calibration curve used to calculate the native molecular weight of Gh-ChrR. Gh-ChrR eluted with a retention time of ∼60 min (solid square), a value that corresponds to an estimated native molecular weight of a tetramer, ∼80 kDa.
(TIF)

Figure S8 **Superposition of Gh-ChrR with EmoB (PDB ID 2VZJ) dimers.** Superposition of one pair of dimers in the tetramer complex formed by Gh-ChrR (magenta) and EmoB (blue). The view in B is a 90° rotation towards the reader about the x-axis. NADH and FMN are highlighted in a stick representation with the carbon atoms of NADH and FMN colored green in EmoB and yellow in Gh-ChrR with all the nitrogen atoms colored blue, oxygen atoms colored red, and phosphorus atoms colored orange.
(TIF)

Figure S9 **Reactive oxygen species (ROS) generated during the reduction of chromate, ferricyanide, and uranyl by Gh-ChrR.** ROS production was monitored using fluorescence probes in a reaction mixture containing 500 µM metal substrate and 100 µM NADH in the presence (grey) and absence (white) of 5 µM Gh-ChrR in buffer containing 50 mM Tris-HCl, 100 mM NaCl, pH 7.4. The experiments were performed in triplicate, at 37°C, with the measurement error shown.
(TIF)

Table S1 **NADH-Dependent Reduction Efficiency of Gh-ChrR for Different Metal Anions^a^**
(DOC)

Table S2 Calculated Substrate Inhibition Kinetics Parameters for Gh-ChrR*
(DOC)
