# Supplementary figures and images for: Structure Determination and Functional Analysis of a Chromate Reductase from Gluconacetobacter hansenii
Source: PLoS One. 2012 Aug 6;7(8):e42432. doi: 10.1371/journal.pone.0042432 (PMC3412864; doi:10.1371/journal.pone.0042432)

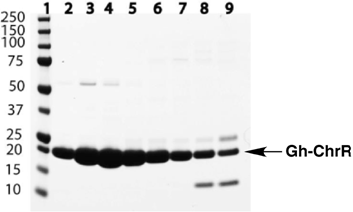

Supplement: Figure S1 — SDS-PAGE analysis of the HiTrap Q fractions containing recombinant Gh-ChrR. Lane1: Molecular weight markers (labeled in kDa on the left). Lanes 2–9: Two µL aliquots from sequential fractions off a HiTrap Q ion exchange column. The major band identified with an arrow is Gh-ChrR (monomeric molecular mass = 21.3 kDa). Fraction 5, free of visible impurities on the gel, was used to crystallize Gh-ChrR and for the enzyme kinetic studies. (TIF) [file pone.0042432.s001.tif]

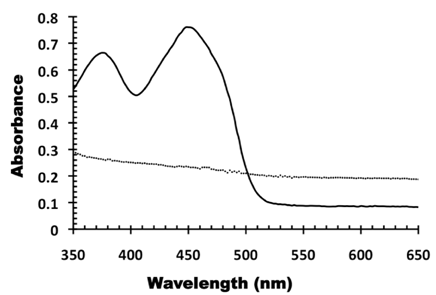

Supplement: Figure S2 — Influence of NADH on the UV/vis absorbance spectrum of Gh-ChrR. Spectra of a freshly purified solution of Gh-ChrR (15 µM protein, 50 mM Tris-HCl, 100 mM NaCl, pH 7.4) recorded before (solid line) and after (dashed line) the addition of excess NADH (100 µM). The spectral changes could be observed visually with the original yellow colored sample turning clear upon the addition of NADH indicating a transition from an oxidized to a reduced state. (TIF) [file pone.0042432.s002.tif]

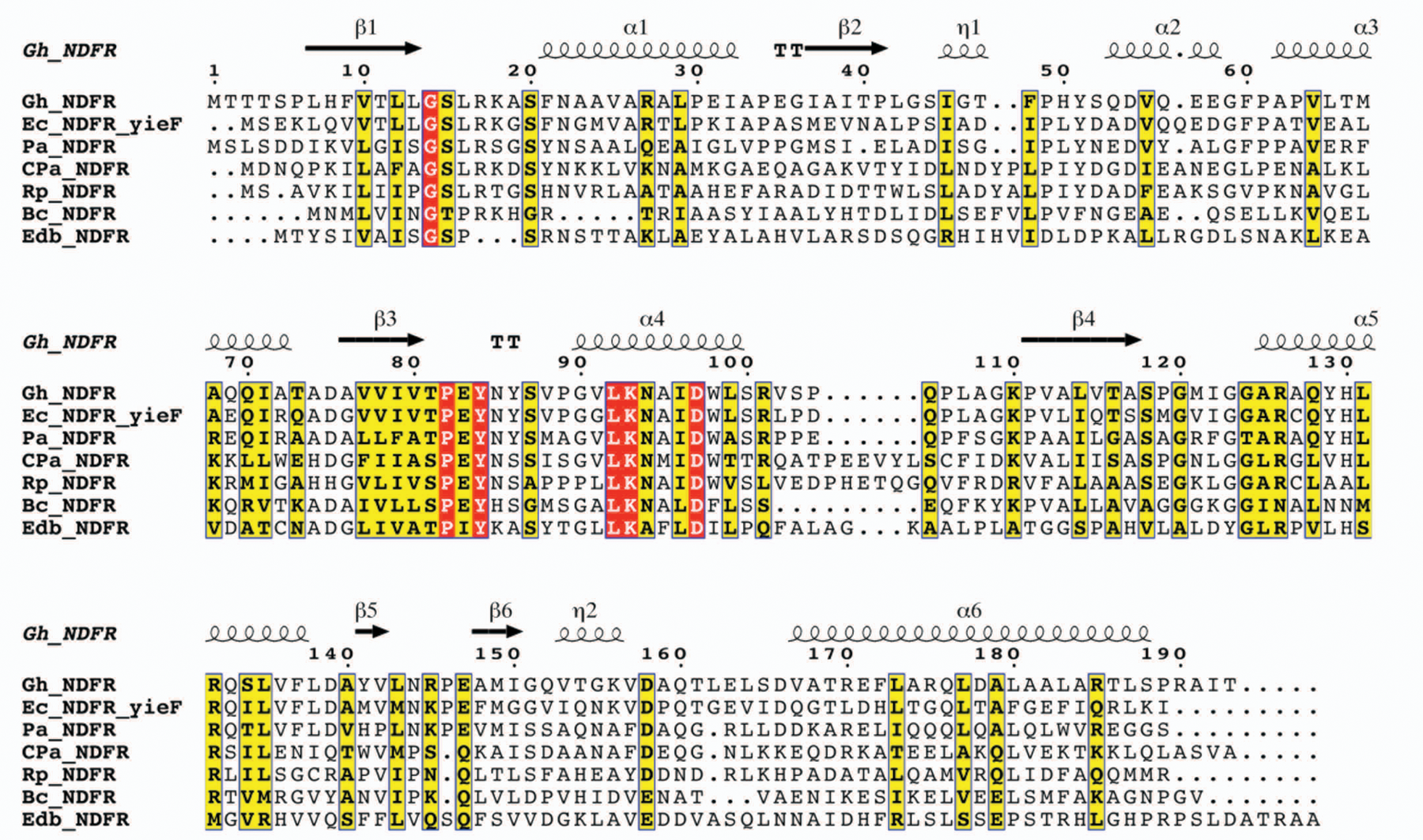

Supplement: Figure S3 — Sequence alignment of Gh-ChrR with other NAD(P)H-dependent FMN reductases (NDFR). The amino acid sequences of the following FMN reductases were aligned using ClustalW2 (http://www.ebi.ac.uk/Tools/msa/clustalw2/): Gh_NDFR: G. hansenii Gh-ChrR [16]; Ec_NDFR: E. coli. IAI39 YieF [63]; Pa_NDFR: P. aeruginosa PAO1 NDFR reductase [26] (PDB entry: 1RTT); Bc_NDFR: B. subtilis str. 168 NDFR [35] (PDB entry: 1NNI); Edb_NDFR: EDTA-degrading bacterium BNC1 EmoB [28] (PDB entry: 2VZJ); Cpa_NDFR: Candidatus Protochlamydia amoebophila UWE25 NDFR [64]; and Rp_NDFR: Rhodopseudomonas palustris NDFR [65]. The secondary structure elements of Gh-ChrR are indicated on the top sequence (310 helices are indicated as η). Identical and conserved residues are highlighted red and yellow, respectively. The elements of secondary structure observed in the crystal structure of Gh-ChrR are shown on top of the alignment. (TIF) [file pone.0042432.s003.tif]

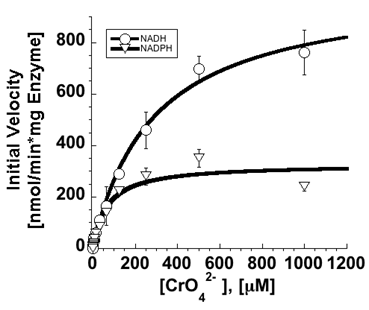

Supplement: Figure S4 — Influence of NADH and NADPH on the rates of chromate reduction by Gh-ChrR. Rate of chromate reduction by Gh-ChrR (5 µM) in the presence 200 uM NADH (open circles) and 200 µM NADPH (open triangles) in buffer containing 50 mM Tris-HCl, 100 mM NaCl, pH 7.4. Experiments were performed in triplicate with error bars for each measurement shown. (TIF) [file pone.0042432.s004.tif]

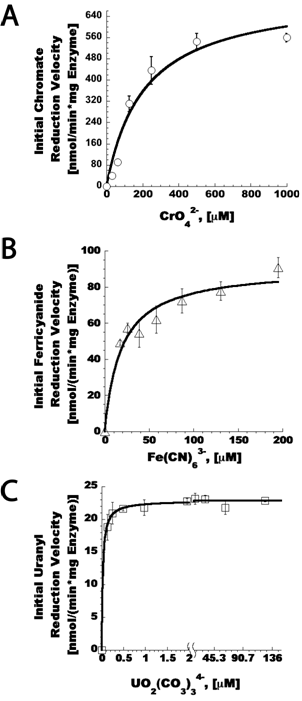

Supplement: Figure S5 — Reduction of chromate, ferricyanide, and uranyl by Gh-ChrR. NADH-dependent reduction rates and associated nonlinear least squares fits (solid lines) for Gh-ChrR (5 µM) in the presence of the indicated concentrations of the metal oxides chromate (A), ferricyanide (B), and uranyl (C). Measurements were made by following NADH consumption and represent the average of triplicate experiments. The kinetic parameters obtained from a nonlinear least-squares fit of this data to Michaelis-Menten equations are listed in Table S1. (TIF) [file pone.0042432.s005.tif]

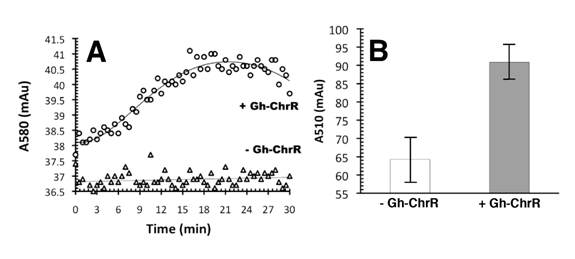

Supplement: Figure S6 — Increase in the levels of Cr(III) and U(IV) following the reduction of Cr(VI) and U(VI), respectively, by Gh-ChrR. A. The chromate reduction product Cr(III) was monitored by the increase in absorbance at 580 nm observed by incubating 125 µM Cr(VI) and 100 µM NADH with (open circles) and without (open triangles) Gh-ChrR. B. The uranyl reduction product uraninite (U(IV)) was monitored by the increase in absorbance at 510 nm observed by incubating 125 µM U(VI) and 100 µM NADH with (white column) and without (grey column) Gh-ChrR. Shown are the average of three independent measurements recorded 20 minutes after the addition of the protein. (TIF) [file pone.0042432.s006.tif]

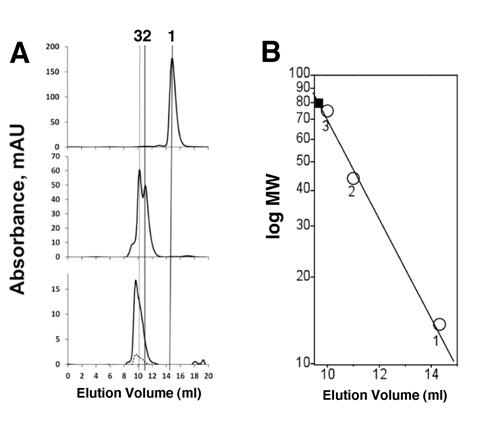

Supplement: Figure S7 — Determination of the native molecular weight of Gh-ChrR by size exclusion chromatography. A. Stacked elution profiles (monitored absorbance at 280 nm) of Gh-ChrR (bottom) and molecular weight standards on a Superdex75 size exclusion column (50 mM Tris-HCl, 100 mM NaCl, pH 7.4): 1. ribonuclease A (13.7 kDa, top panel); 2. ovalbumin (44 kDa, middle panel); and 3. conalbumin (75 kDa, middle panel). The dashed line in the bottom chromatogram is the absorbance at 340 nm corroborating that FMN was bound to the protein. B. The calibration curve used to calculate the native molecular weight of Gh-ChrR. Gh-ChrR eluted with a retention time of ∼60 min (solid square), a value that corresponds to an estimated native molecular weight of a tetramer, ∼80 kDa. (TIF) [file pone.0042432.s007.tif]

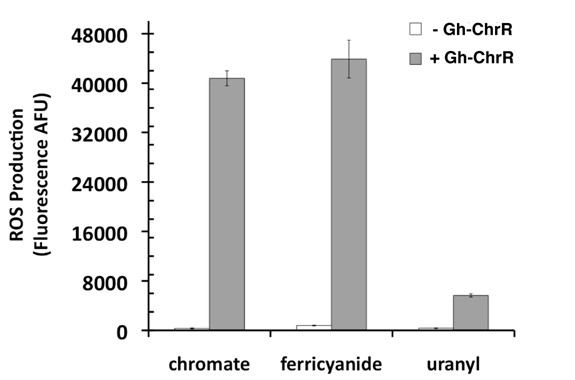

Supplement: Figure S9 — Reactive oxygen species (ROS) generated during the reduction of chromate, ferricyanide, and uranyl by Gh-ChrR. ROS production was monitored using fluorescence probes in a reaction mixture containing 500 µM metal substrate and 100 µM NADH in the presence (grey) and absence (white) of 5 µM Gh-ChrR in buffer containing 50 mM Tris-HCl, 100 mM NaCl, pH 7.4. The experiments were performed in triplicate, at 37°C, with the measurement error shown. (TIF) [file pone.0042432.s009.tif]
