# Supplementary material for: Structure Determination and Functional Analysis of a Chromate Reductase from Gluconacetobacter hansenii
Source: PLoS One. 2012 Aug 6;7(8):e42432. doi: 10.1371/journal.pone.0042432 (PMC3412864; doi:10.1371/journal.pone.0042432)
Supplement: Table S1 — NADH-Dependent Reduction Efficiency of Gh-ChrR for Different Metal Anionsa (DOC) [file pone.0042432.s010.doc]

**Table S1**

**NADH-Dependent Reduction Efficiency of Gh-ChrR for Different Metal Anions^a^**

| **Substrates** | **V_max_**  **[nmol/(min mg enzyme)]** | **Apparent**  ***K_m_* (μM) ^a^** | **Apparent**  ***k_cat_***  **(s^-1^) ^a^** | **Apparent**  ***k_cat_/K_m_***  **(M^-1^ s^-1^)^b^** |
| --- | --- | --- | --- | --- |
| Chromate | 750 ± 90 | 240 ± 70 | 0.25 ± 0.03 | 1,000 ± 300 |
| Ferricyanide | 91 ± 6 | 19 ± 5 | 0.030 ± 0.002 | 1,600 ± 300 |
| Uranyl | 23.0 ± 0.2 | < 0.1 | 0.008 ± 0.0008 | >80,000 |

**^a^** Values are based on triplicate measurements, where steady-state kinetic data for Gh-ChrR (5 μM) were measured at a constant NADH concentration (100 μM) and fit to the Michaelis-Menten equation.

**^b^** Calculations based on a molecular mass of tetrameric Gh-ChrR, 80kDa, and four independent active sites.
