# Supplementary material for: Structure Determination and Functional Analysis of a Chromate Reductase from Gluconacetobacter hansenii
Source: PLoS One. 2012 Aug 6;7(8):e42432. doi: 10.1371/journal.pone.0042432 (PMC3412864; doi:10.1371/journal.pone.0042432)
Supplement: Table S2 — Calculated Substrate Inhibition Kinetics Parameters for Gh-ChrR* (DOC) [file pone.0042432.s011.doc]

**Table S2**

**Calculated Substrate Inhibition Kinetics Parameters for Gh-ChrR***

| **Kinetic Parameters** | **Calculated values** |
| --- | --- |
| *K_i_* (NADH) | 3.5 ± 0.7 μM |
| *K_ia_* (CrO_4_^2-^) | 6 ± 2 μM |
| *K_mA_* (CrO_4_^2-^) | 5 ± 1 μM |
| *K_mB_* (NADH) | 7 ± 1 μM |

* Assumes substrate inhibition model involving ordered bireactant substrate binding (see Figure 1), where kinetic constants (*K_i_, K_ia_, K_mA_, K_mB_*) are calculated as described in the Experimental Procedures.
